# Improvement of diaphragm and limb muscle isotonic contractile performance by K^+ ^channel blockade

**DOI:** 10.1186/1743-0003-7-1

**Published:** 2010-01-11

**Authors:** Erik van Lunteren, Jennifer Pollarine

**Affiliations:** 1 Division of Pulmonary & Critical Care Medicine, Louis Stokes Cleveland Department of Veterans Affairs Medical Center and Case Western Reserve University, Cleveland, OH 44106, USA

## Abstract

The K^+ ^channel blocking aminopyridines greatly improve skeletal muscle isometric contractile performance during low to intermediate stimulation frequencies, making them potentially useful as inotropic agents for functional neuromuscular stimulation applications. Most restorative applications involve muscle shortening; however, previous studies on the effects of aminopyridines have involved muscle being held at constant length. Isotonic contractions differ substantially from isometric contractions at a cellular level with regards to factors such as cross-bridge formation and energetic requirements. The present study tested effects of 3,4-diaminopyridine (DAP) on isotonic contractile performance of diaphragm, extensor digitorum longus (EDL) and soleus muscles from rats. During contractions elicited during 20 Hz stimulation, DAP improved work over a range of loads for all three muscles. In contrast, peak power was augmented for the diaphragm and EDL but not the soleus. Maintenance of increased work and peak power was tested during repetitive fatigue-inducing stimulation using a single load of 40% and a stimulation frequency of 20 Hz. Work and peak power of both diaphragm and EDL were augmented by DAP for considerable periods of time, whereas that of soleus muscle was not affected significantly. These results demonstrate that DAP greatly improves both work and peak power of the diaphragm and EDL muscle during isotonic contractions, which combined with previous data on isometric contractions indicates that this agent is suitable for enhancing muscle performance during a range of contractile modalities.

## Background

The aminopyridines are a group of agents which block membranous K^+ ^channels in excitable tissues such as neurons and skeletal muscle [1,2]. Their major electrophysiological effect is to slow the rate of action potential repolarization, thereby prolonging action potential duration and increasing the depolarization-time integral (area under the curve of the action potential) [3-5]. In skeletal muscle the action potential prolongation increases calcium influx [6] and augments isometric force at low to intermediate (but not high) stimulation frequencies [3,4,7-10]. The aminopyridines (in particular 3,4-diaminopyridine, or DAP) have been used for treating human diseases such as Lambert-Eaton myasthenic syndrome [11-14].

The lack of force increase produced by the aminopyridines at high stimulation frequencies [8,15] potentially limits their clinical utility for generalized muscle weakness due to aging or disease. However, during functional neuromuscular stimulation applications designed to restore motor activity in subjects with spinal cord injuries, low to intermediate rather than high stimulation frequencies are the rule [16,17]. Some restorative applications are currently limited by the need to generate high force values while at the same time avoiding muscle fatigue, in particular for weight bearing activities such as standing up from a seated position, maintaining a standing posture, and walking. A number of electrical stimulation paradigms have been devised to optimize the input-output relationship of skeletal muscle, such as variable frequency stimulation [18-22], but this has had limited clinical effectiveness in human functional neuromuscular stimulation applications. A potential limitation of this strategy is that the force increases are relatively modest, in particular when compared with the force augmentation that can be achieved pharmacologically with DAP [9].

The inotropic effects on skeletal muscle of DAP and other aminopyridines has been studied extensively under isometric contractile conditions, during which there is force generation without shortening. Findings in normal rat diaphragm muscle for DAP include twitch force increases of ~70 to 180% (depending on age, exercise status and strain), a large left-ward shift of the force-frequency relationship, good maintenance of force increases during fatigue-inducing stimulation, and variable prolongations of isometric contraction and half-relaxation times [4,8-10,23]. Limited data directly examining the effects of DAP [10] and other aminopyridines [24-26] suggest heterogeneity of contractile improvements for muscles with different slow *vs *fast fiber type composition when assessed under isometric conditions.

Many functional tasks involve a combination of non-shortening and shortening contractions, often with different muscles performing one type or the other, but in some instances with one muscle engaging in both types of contractions during different phases of the task [27,28]. Isometric and isotonic contractions differ from each other with regards to actin-myosin cross-bridge formation and cellular energetics. As a result, information about DAP effects on contractile performance under isometric conditions can not be extrapolated to isotonic conditions, especially during the course of repetitive fatigue-inducing contractions. The hypothesis of the present study is that DAP improves the isotonic contractile performance of skeletal muscles, but in a non-uniform manner among skeletal muscles.

## Methods

All studies were approved by the Institutional Animal Care and Use Committee and complied with NIH animal care guidelines. Seventeen Sprague-Dawley rats obtained from Charles Rivers (Wilmington, MA) were studied when they weighed 338 ± 15 g. Rats were anesthetized with rodent anesthesia cocktail (initial dose, ketamine 21-30 mg/kg, xylazine 4.3-6.0 mg/kg and acepromazine 0.7-1.0 mg/kg, with supplemental smaller doses given as needed to produce and maintain a deep level of anesthesia). Soleus, extensor digitorum longus (EDL), and diaphragm were removed surgically. Muscles were initially placed in aerated (95% O_2_-5% CO_2_) physiological solution which was kept at room temperature. The composition of the physiological solution was consistent with previous studies (in mM) [4,8-11,22]: NaCl 135, KCl 5, CaCl2 2.5, MgSO4 1, NaH2PO4 1, NaHCO3 15, glucose 11, with the pH adjusted to 7.35-7.45. The diaphragm was cut into strips that were ~3 mm wide, whereas EDL and soleus muscles were kept intact and not cut. Special care was taken to keep the tendinous and bony origins and insertions of each muscle sample intact. The muscle samples were subsequently mounted vertically in a double-jacketed bath containing physiological solution kept at a constant 37°C which was aerated (95% O_2_-5% CO_2_) continuously. Muscles were attached to a transducer (model 305, Aurora Scientific, Ontario, Canada). This dual-mode servo-controlled force transducer measured force and length separately, and held force constant while changes in length were measured. The muscle strips underwent electrical stimulation with a pulse width of 1 msec [4,8] via parallel platinum electrodes placed ~4 mm apart with the muscle situated in the middle [4,8-10]. Supramaximal voltages were used; voltage was increased until there was no further increase in the magnitude of the contraction, and then an additional 20% was added to this value [4,8-10]. All muscle strips were tested at optimal length (L_o_) based on twitch force. In a previous study of isometric contractions using the same *in vitro *approach we have found for diaphragm, soleus and EDL that force of muscles incubated with no drug were stable over 20 minutes (which is similar to the time needed for the present studies) and, furthermore, DAP effects could easily be discerned relative to force values of muscle samples that were not treated with drug [[10], and unpublished data].

The study consisted of two parts, a) delineation of DAP effects on isotonic contractile performance as a function of load when stimulated at 20 Hz, and b) determination of the extent to which DAP improves isotonic contractions over time during fatigue-inducing stimulation. Separate muscle samples were used for each part of the study. The DAP concentration used throughout was 0.3 mM, which was chosen because it was the lowest amount that resulted in a near-maximal force increase in rat diaphragm muscle [8] and has been used for several subsequent diaphragm isometric studies [4,9,10]. In addition, in a study comparing isometric contractions of diaphragm, soleus and EDL, a concentration of 0.3 mM resulted in the maximum force increase that was sustained over time for all three muscles [10]. A stimulation frequency of 20 Hz was chosen for both portions of the present study, based on DAP and other aminopyridines improving isometric force at low to intermediate (~1 to 50 Hz) but not high stimulation frequencies [8,24,29], and that previous studies of DAP effects on isometric fatigue in rat muscle used this stimulation frequency [4,8,10,23], thereby facilitating comparisons of isotonic with previous isometric data.

In order to assess DAP effects on isotonic contractions as a function of load, muscles were stimulated for 333 msec at seven different loads (5, 10, 20, 30, 40, 50 and 60% load) with a minute of no stimulation in between each load so as to prevent fatigue. DAP (0.3 mM) or additional physiological solution was incubated for 10 min before the seven loads were tested again. Comparisons were made for the post-DAP versus post-no drug data to factor out the effects of repeated stimulation. The loads for all parts of the study were percentages of maximum force during 20 Hz stimulation before the addition of DAP or no drug. The choice of using peak force during 20 Hz stimulation rather than tetanic force to define maximum load was based on two considerations. First, it is consistent with the approach used in our previous studies of muscle isotonic contractile properties [30,31]. Second, the present study was performed in the context of functional electrical stimulation, and thus it is more meaningful to base loads on force produced during the frequency at which the muscle will be stimulated.

Muscle fatigue was tested at a single load of 40% for all muscles. The load of 40% was chosen because it yielded approximately maximum work for all three muscles. Separate samples were tested in the absence and presence of DAP, so that drug and no-drug data were obtained from muscle samples which underwent identical stimulation paradigms. For fatigue testing, muscles were stimulated at 20 Hz using a train duration of 333 ms, with one train every 2 sec. Muscle length always returned to baseline in between stimulus trains, allowing total shortening and maximum velocity of shortening to be calculated for each stimulus train. Changes in contractile parameters were measured over time. To factor out DAP effects on contractile parameters at the onset of stimulation, a fatigue index was calculated as the contractile parameter at the end of 2 minutes of stimulation relative to the initial value.

Data were relayed to a computer using the data acquisition and analysis program Dynamic Muscle Control (Aurora Scientific Inc., Ontario, Canada). Muscle performance was evaluated by measuring work and power. Work was calculated as the product of the isotonic afterload and the total amount of shortening during each train (the difference between muscle length when not stimulated and the maximum amount of shortening that occurred during the train). Peak power was calculated as the product of the isotonic afterload and shortening velocity, with velocity measured during the early portion of the contraction when it was at or near its maximal value for each train [30-32].

Data were analyzed statistically using 2-way RMANOVA; for the load curves the factors were load and DAP treatment, whereas for fatigue testing the factors were duration of stimulation and DAP treatment. RMANOVA was followed with the Newman-Kuels test when significance was found to evaluate the effects of DAP treatment. Twitch contraction and fatigue index data were analyzed with paired and unpaired t tests, respectively. Probability values of P ≤ 0.05 were considered to be statistically significant. Data appear as mean values ± 1 SE.

## Results

### 20 Hz Contractions at Various Loads

**Figure 1:**
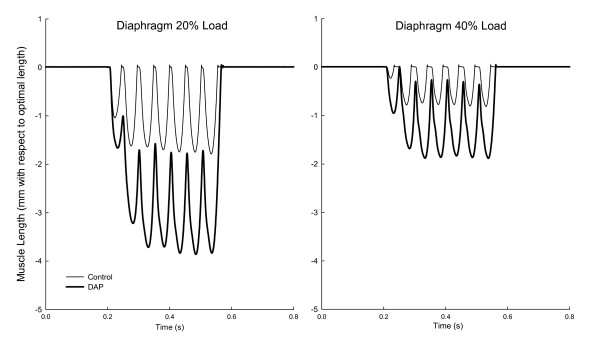
**Examples of diaphragm isotonic shortening at two different loads in the presence and absence of 3,4-diaminopyridine (DAP)**. Optimal length of this muscle sample was 21 mm.

**Figure 2:**
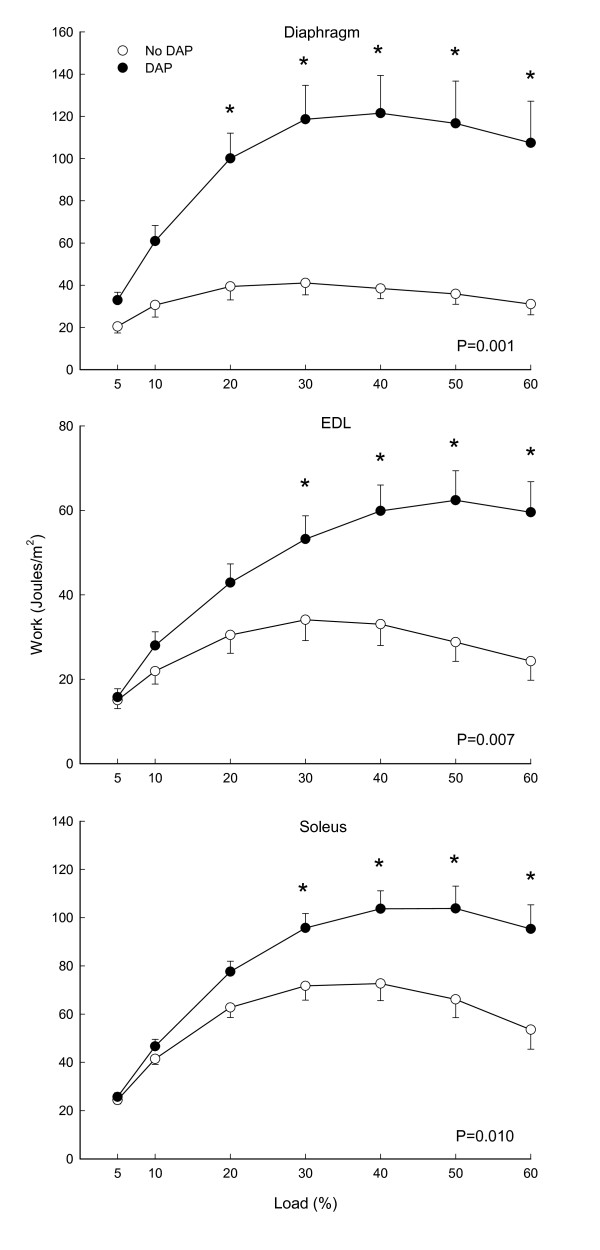
**Effects of 3,4-diaminopyridine (DAP) on isotonic work of diaphragm, extensor digitorum longus (EDL) and soleus as a function of load during 20 Hz stimulation**. P values indicate results of 2-way RMANOVA testing for each panel, and asterisks (*) indicate significant differences at each load per the Newman-Kuels test.

**Figure 3:**
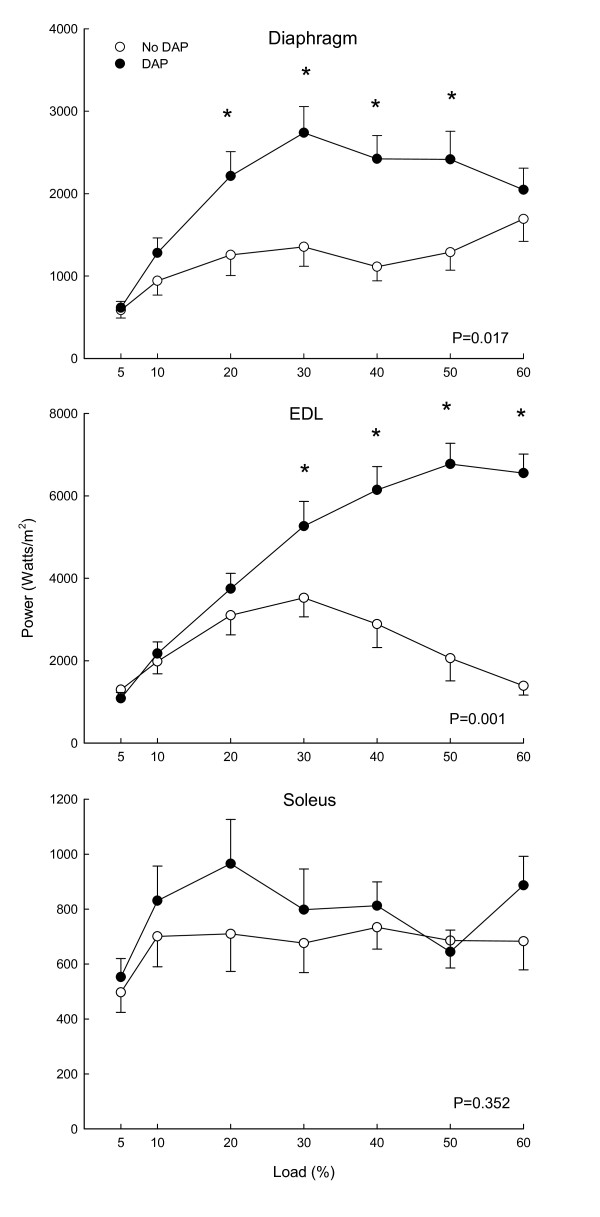
**Effects of 3,4-diaminopyridine (DAP) on peak isotonic power of diaphragm, extensor digitorum longus (EDL) and soleus as a function of load during 20 Hz stimulation**. P values indicate results of 2-way RMANOVA testing for each panel, and asterisks (*) indicate significant differences at each load per the Newman-Kuels test.

An example of muscle length tracings of the diaphragm during isotonic contractions is depicted in Figure 1, demonstrating representative increases in muscle shortening by DAP at two loads. Work was increased by DAP for the diaphragm (P = 0.001), EDL (P = 0.007) and soleus (P = 0.01) muscles (Figure 2). For the diaphragm the increase was significant at loads ranging from 20 to 60%, whereas for the EDL and soleus the increases were significant at loads of 30 to 60%. The effects of DAP on peak power, however, were more variable among muscles (Figure 3), increasing significantly for the diaphragm (P = 0.017) and EDL (P = 0.001) but not for the soleus (P = 0.35). For the diaphragm peak power was increased at loads of 20 to 50%, whereas EDL power was increased significantly at loads of 30 to 60%. In contrast, peak power was not significantly increased for the soleus muscle at any load.

### Fatigue During Repetitive Contractions

**Figure 4:**
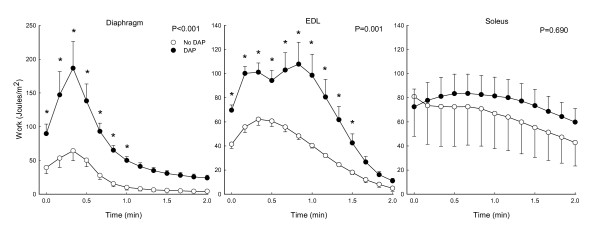
**Effects of 3,4-diaminopyridine (DAP) on changes in isotonic work of diaphragm, extensor digitorum longus (EDL) and soleus during repetitive 20 Hz stimulation at a load of 40%**. P values indicate results of 2-way RMANOVA testing for each panel, and asterisks (*) indicate significant differences at each load per the Newman-Kuels test.

**Figure 5:**
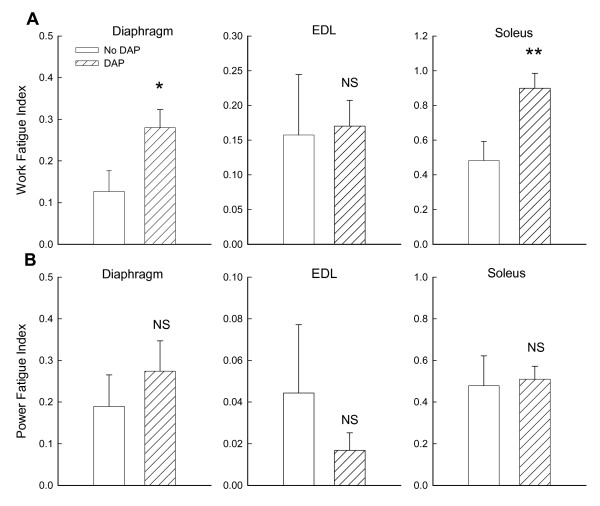
**Effects of DAP on fatigue indexes for isotonic work (A) and peak power (B) of diaphragm, extensor digitorum longus (EDL) and soleus during repetitive 20 Hz stimulation at a load of 40%**. Asterisks indicate significant increases: ** P ≤ 0.01, * P < 0.05, NS = not significant.

For the diaphragm, there was a brisk initial increase in work near the onset of repetitive stimulation, which was found both in the absence and presence of DAP (Figure 4). However, the magnitude of the early work increase was augmented by DAP. The initial increase was followed by progressive declines in work for both untreated and DAP-treated muscle. Nonetheless, work of DAP-treated muscle was significantly greater than that of untreated muscle (P < 0.001), in particular for the first half of the fatigue testing period. Furthermore, the fatigue index for work was higher in DAP-treated than untreated muscle (indicating a smaller relative drop in work over time with DAP) (Figure 5A). For the EDL, the transient work increase at the beginning of stimulation was both increased and prolonged by DAP, and work was augmented by DAP (P = 0.001) for most of the repetitive stimulation period (Figure 4). However in contrast to the diaphragm, the work fatigue index was similar in the presence and absence of DAP (Figure 5A). Work of the soleus muscle over time was not affected by DAP (P = 0.69) (Figure 4), although the fatigue index was higher in DAP-treated than untreated muscle (Figure 5A).

**Figure 6:**
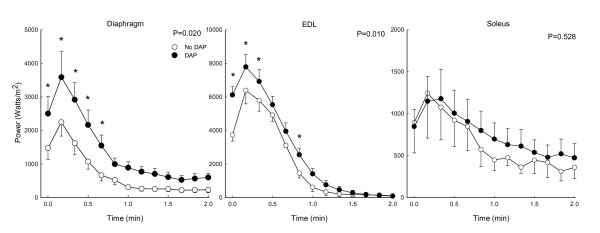
**Effects of 3,4-diaminopyridine (DAP) on changes in isotonic peak power of diaphragm, extensor digitorum longus (EDL) and soleus during repetitive 20 Hz stimulation at a load of 40%**. P values indicate results of 2-way RMANOVA testing for each panel, and asterisks (*) indicate significant differences at each load per the Newman-Kuels test.

Peak power of the diaphragm was also augmented by DAP during fatigue-inducing stimulation (P = 0.02) (Figure 6). This was also the case for the EDL (P = 0.01), although the magnitude and duration of the increases were generally smaller than for the diaphragm. However, the fatigue index for peak power was not altered by DAP for either diaphragm or EDL (Figure 5B). DAP did not affect peak power of the soleus muscle over time (P = 0.53) nor did it affect the soleus muscle power fatigue index.

## Discussion

The major finding of the present study was that DAP can substantially improve the isotonic contractile performance of skeletal muscle during contractions elicited by 20 Hz stimulation, albeit to a non-uniform extent among skeletal muscles. For the diaphragm and EDL muscles work and peak power were augmented during contractions over a range of loads, and furthermore these augmentations persisted over time during fatigue-inducing repetitive stimulation when tested at a single load (of 40%). In contrast, the beneficial effects of DAP on soleus muscle isotonic contractile performance were much more limited, and were noted for work (and thus for extent of shortening) but not for peak power (and thus not for peak velocity of shortening).

Most isometric data for DAP have been obtained with diaphragm muscle [4,8-10,15,23], and we will therefore initially focus on diaphragm data from the present study for comparisons of current isotonic and previous isometric data. The first conclusion from such comparisons is that DAP improves diaphragm performance over a range of loading conditions, ranging from small to intermediate loads in which there is considerable shortening (present study) to very large loads which prevent shortening altogether (previous isometric studies). The second conclusion is that the magnitude of the improved diaphragm contractile performance with DAP is large for both isotonic and isometric contractions. As noted in the introduction, the magnitude of isometric twitch force increases for the diaphragm is in the range of 70 to 180%. Values for diaphragm twitch force increases from three studies in sedentary young adult Sprague Dawley rats (similar to those used in the present study) averaged 111%, and the isometric force increases during 20 Hz stimulation were similar in size [8,10,23]. In the present study, DAP-induced increases in diaphragm work and peak power during isotonic contractions varied as a function of load (Figures 1, 2, 3). Nonetheless, improvements in isotonic contractile parameters were in many instances as large as the force increases found during isometric contractions. A third conclusion is that DAP-induced increases in diaphragm contractile performance are well-maintained over the course of fatigue-inducing repetitive stimulation during both isometric (previous studies) and isotonic (Figures 4, 5, 6) contractions. In the present study during isotonic contractions work and power was significantly elevated by DAP for the first 40-60 seconds of a two minute repetitive stimulation period, and contractile performance of DAP-treated muscle never declined below that of untreated muscle through the two minutes of stimulation. This is comparable to the 30-80 second duration of isometric force improvement by DAP found during previous *in vitro *studies of normal rat diaphragm muscle [4,8,10,22].

There are several studies which have compared the effects of aminopyridines on the isometric contractile performance of different muscles, although most studies used 4-aminopyridine rather than DAP. It should be kept in mind that 4-aminopyridine produces smaller force increases and lesser degrees of action potential prolongation than DAP [4,5,8,10,24,33]. Only four studies compared responses of different muscles directly. The first found that 4-aminopyridine improved twitch force of the tibialis anterior muscle but not the soleus muscle [26]. The second study found similar force increases for rat diaphragm (64%) and sternohyoid muscle (55%) in response to 4-aminopyridine [24]. The third found that 4-aminopyridine increased rat diaphragm twitch force to a greater extent (71 ± 7%) than that of two limb muscles, the extensor digitorum longus (28 ± 11%) and the soleus muscle (22 ± 3%) [25]. The most recent study found that DAP-induced force increases were greater for diaphragm and EDL than soleus, but that the force increases were maintained for a longer time for soleus than diaphragm than EDL [10]. Thus isometric data paint a picture of considerable diversity among muscles in the degree to which contractile performance is altered by the aminopyridines, with which the present study is in agreement.

The present study used a single stimulation frequency (20 Hz) for all three muscles. This frequency differs among muscles in terms of how this relates to their natural motor unit firing frequencies during normal behaviors in the intact animal, with faster muscles such as the EDL being activated normally at higher frequencies than slower muscles such as the soleus [34], as well as how it relates to their force-frequency relationships, with 20 Hz causing greater degree of contractile fusion in slower muscles such as the soleus compared with faster muscles such as the EDL. DAP and other aminopyridines prolong action potential duration [3-5] thereby increasing calcium influx [6] and enhancing muscle contraction [3,4,7-10]. Thus DAP-treated muscle stimulated at a low frequency of stimulation should achieve the same intracellular calcium concentrations and hence force production as untreated muscle stimulated at a higher stimulation frequency - and this is borne out by data on force-frequency relationships of untreated and DAP-treated muscle studied during isometric contractions [8]. During 20 Hz stimulation (without DAP), soleus contractions are already quite fused and thus the additional degree of fusion with DAP does not augment shortening much if at all; at 20 Hz (without DAP) diaphragm contractions are right at the threshold of being fused (see in particular left panel of Figure 1) and thus DAP enhances fusion a lot and thus increases muscle shortening considerably; and at 20 Hz (without DAP) EDL contractions are further away than the diaphragm from the fusion threshold, and thus DAP produces a more modest amount of fusion and thus a smaller augmentation of muscle shortening. There may also be other mechanisms in addition to the above accounting for differences among muscles in DAP effects. There are multiple types of K^+ ^channels, including multiple subtypes of delayed rectifier K^+ ^channels, in skeletal muscle, and various channel types and subtypes may have differential sensitivity to aminopyridines including DAP. It is possible (albeit speculative) that the three muscles studied have different proportions of various K^+ ^channel types and subtypes, with the diaphragm having the highest proportion of K^+ ^channel subtypes with high DAP sensitivity.

## Conclusions

The aminopyridines have been used for treating human diseases such as Lambert-Eaton myasthenic syndrome, with DAP being preferred over 3,4-aminipyridine due to reduced crossing of the blood-brain barrier and thus lower propensity to cause neurological side effects [11-14]. The present data, combined with previous isometric studies, have several implications for the potential clinical use of DAP to augment skeletal muscle contractile performance during functional neuromuscular stimulation applications. First is that DAP appears to be effective over a range of loads, and therefore suitable for both isometric and isotonic (and presumably also mixed) restorative applications. Second is that the DAP-induced contractile augmentations can be maintained over time during repetitive fatigue-inducing stimulation under both isotonic and isometric conditions. It should be noted in this regard that fatigue occurs much more rapidly with *in vitro *than *in vivo *muscle preparations [10,15], so that it is quite possible that the contractile augmentations *in vivo *will be longer lasting than those depicted in the present study. Not yet known is whether DAP affects recovery from fatigue and whether the salutatory effects of DAP on contractile performance would be equally large during a second set of contractions following a recovery period as it had been during the initial set of contractions. Third is that one should expect differences among skeletal muscles in the degree of inotropic effects provided by DAP during both isotonic and isometric contractions, which may in part be influenced by the stimulation frequency used for muscle activation relative to the normal activation rates of each muscle when activated by the brain in vivo as well as the force-frequency relationships of each muscle. Variability among muscles might be less of an issue for spinal cord injury subjects, in that the upper motoneuron denervation results in all affected muscles acquiring a fast-contraction and fast-myosin phenotype. On the other hand, muscles typically undergo a reconditioning paradigm as part of functional neuromuscular stimulation programs. This results in a movement towards a slower phenotype, and it is possible that DAP effects may therefore change during the course of the reconditioning program. On the other hand, a complete conversion to a slow phenotype is typically not produced by the reconditioning programs used for limb and diaphragm muscle restorative applications (in contrast to cardiomyoplasty applications), and both previous isometric studies and the present isotonic study indicate that a mixed muscle such as the diaphragm responds nicely to DAP by increasing force, peak power and work.

## List of Abbreviations

DAP: 3,4-diaminopyridine; EDL: extensor digitorum longus.

## Competing interests

The authors declare that they have no competing interests.

## Authors' contributions

EvL conceived of the study, participated in the design of the study, participated in the data analysis, and participated in writing the manuscript. JP participated in the design of the study, carried out the contractile studies, performed the statistical analysis, and participated in writing the manuscript. All authors read and approved the final manuscript.
